# Structural MRI Differences between Patients with and without First Rank Symptoms: A Delusion?

**DOI:** 10.3389/fpsyt.2015.00107

**Published:** 2015-07-29

**Authors:** Henriette D. Heering, Godefridus J. C. Koevoets, Laura Koenders, Marise W. J. Machielsen, Carin J. Meijer, Manabu Kubota, Jessica de Nijs, Wiepke Cahn, Hilleke E. Hulshoff Pol, Lieuwe de Haan, Rene S. Kahn, Neeltje E. M. van Haren

**Affiliations:** ^1^Department of Psychiatry, Academic Medical Centre, Amsterdam, Netherlands; ^2^Brain Centre Rudolf Magnus, University Medical Centre Utrecht, Utrecht, Netherlands

**Keywords:** schizophrenia, first rank symptoms, structural MRI image analysis, subcortical volume, corticalvolumes

## Abstract

**Objective:**

It has been suggested that specific psychotic symptom clusters may be explained by patterns of biological abnormalities. The presence of first rank symptoms (FRS) has been associated with cognitive abnormalities, e.g., deficits in self-monitoring or in the experience of agency, suggesting that a specific network of neural abnormalities might underlie FRS. Here, we investigate differences in cortical and subcortical brain volume between patients with and without FRS.

**Methods:**

Three independent patient samples (referred to as A, B, and C) with different mean ages and in different illness stages were included, leading to a total of 348 patients within the schizophrenia-spectrum. All underwent magnetic resonance imaging of the brain. In addition, the presence of FRS was established using a diagnostic interview. Patients with (FRS+, A: *n* = 63, B: *n* = 129, and C: *n* = 96) and without FRS (FRS−, A: *n* = 35, B: *n* = 17, and C: *n* = 8) were compared on global and local cortical volumes as well as subcortical volumes, using a whole brain (cerebrum) approach.

**Results:**

Nucleus accumbens volume was significantly smaller in FRS+ as compared with FRS− in sample A (*p* < 0.005). Furthermore, FRS+ showed a smaller volume of the pars-opercularis relative to FRS− in sample B (*p* < 0.001). No further significant differences were found in cortical and subcortical volumes between FRS+ and FRS− in either one of the three samples after correction for multiple comparison.

**Conclusion:**

Brain volume differences between patients with and without FRS are, when present, subtle, and not consistent between three independent samples. Brain abnormalities related to FRS may be too subtle to become visible through structural brain imaging.

## Introduction

Early in the twentieth century, Kurt Schneider introduced the concept of *first rank symptoms* (FRS). He identified certain symptoms as being characteristic of schizophrenia and therefore exhibiting a “first-rank” status in the hierarchy of potentially diagnostic symptoms. These symptoms are auditory hallucinations, passivity experiences (delusions of control), thought withdrawal, thought insertion, thought broadcasting, and delusional perception. FRS are indeed not uncommon in patients with a diagnosis in the psychosis spectrum, but incidence estimates are highly inconsistent. Studies show that between 55 and 84% of patients suffering from psychosis (or schizophrenia, depending on the inclusion criteria) experience at least one first rank symptom during the course of illness (1–4). Consequently, over the last decades the concept has lost its diagnostic applicability (5). One of the reasons is accumulating evidence showing that FRS are not only distinctive for schizophrenia but are also present in patients with bipolar disorder or other non-schizophrenic psychoses (3, 5–7). However, for research purposes, a well-defined cluster of symptoms may help unravel the underlying mechanisms of psychosis. Indeed, studying symptom clusters within the psychosis spectrum might lead to more homogeneous groups of individuals who share a unique biological signature (8).

A model that explains the presence of FRS in terms of a specific pattern of biological or cognitive abnormalities is the motor-prediction model (9). This neurocognitive model suggests that schizophrenia patients are unable to distinguish between actions that are brought about by external forces and those that are generated internally. Prediction of action consequences is crucial in order to self-monitor actions and ascribe agency over actions to self or others. The model argues that an internal copy of a movement-producing signal as generated by the motor system, a so-called *efference copy*, is compared with the actual sensory signals that result from the action. Agency over own actions arises when the efference copy and the sensory feedback matches. Evidence suggests that psychotic patients with FRS show abnormalities in such predictions, consequently leading to mismatches between the actual sensation with the predicted sensation leading to the interpretation of externally generated movements or thought (10). Indeed, cognitive studies on agency provide evidence for deficits in the prediction of sensory consequences of one’s actions, consequently leading to a disturbed sense of agency (10–12). A disconnection between frontal and parietal areas has been suggested to underlie deficits in motor prediction. In healthy individuals, the frontal cortex is assumed to initiate actions while parietal regions represent the current and predicted state of limbs (9).

Studies using Magnetic Resonance Image (MRI) scans of schizophrenia patients have shown associations between FRS and reduced brain volume in the inferior partial lobule (13–15), however, not exclusively. The presence of FRS has also been inversely associated with gray matter volumes of the para-hippocampal gyrus, frontal cortex, cingulate gyrus, basal ganglia, and thalamus (16, 17). In contrast, earlier MRI studies found no associations between FRS and brain volume (18), leaving doubt whether FRS are related to abnormal structural brain measures. In the current study, we aimed to investigate differences in cortical and subcortical volumes between patients with and without first rank symptoms. We choose to take a whole brain (cerebrum) approach and not to focus on predetermined regions of interest (ROIs), due to the discrepancy in ROIs found so far. We included three independent samples with patients in different stages of the disease in order to replicate potential findings across these samples.

## Materials and Methods

### Clinical assessment

Three independent samples were included consisting of patients with affective and non-affective psychosis. In all samples, diagnosis and presence and severity of symptoms were based on the Comprehensive Assessment of Symptoms and History interview [CASH; (19)]. This diagnostic tool is developed to provide information about the current and past symptoms of psychosis in the affective and schizophrenia spectrum. The CASH was administered by experienced clinicians. In Sections 6 and 7 of the CASH, symptom type and severity of positive psychotic symptoms were assessed on both a life-time (dichotomous) and a present state scale (six-point Likert scale from 0 = absent to 5 = severe). Section 6 describes delusions, i.e., paranoid, jealousy, guilt, grandiosity, religious, somatic, reference, alien body control, and abnormal perception phenomena, such as thought reading, thought broadcasting, thought insertions, and thought withdrawal. The latter five symptoms are defined as part of first rank delusions (FRD). Section 7 describes auditory hallucinations (including audible thoughts), voices commenting and conversational voices, somatic, tactile, olfactory, and visual hallucinations. The first three are considered first rank hallucinations (FRH). The presence of either FRD or FRH represented the presence of FRS (19).

In addition, the Positive and Negative Syndrome Scale (PANSS) was administered in all three samples. The PANSS is a 30-item rating scale with three subscales representing positive, negative, and general psychopathology symptoms. Each symptom is rated on a seven-point scale (1 = absent to 7 = extremely severe) (20). In sample A, only the PANSS remission criteria were administered, i.e., remission is achieved when eight symptoms do not exceed a score of mild (severity criterion). These eight core symptoms together represent the psychotic, disorganized, and negative symptom dimensions of schizophrenia (21).

### Subjects

In this study, we included three independent patient samples. Sample A was recruited in Amsterdam, samples B and C were recruited in Utrecht. The ethics committee of Academic Medical Center in Amsterdam (AMC) and UMC Utrecht approved this study.

#### Sample A

A total of 98 patients with a psychotic disorder (schizophrenia: *n* = 68, schizophreniform disorder: *n* = 10, schizoaffective disorder: *n* = 15, substance induced psychotic disorder: *n* = 2, psychosis *not other specified*: *n* = 3) were clinically obtained from the Early Psychosis Department of the AMC between June 2004 and December 2011. This department is specialized in treating young adults with recent-onset psychotic disorders. Patients had a mean age of 22.5 years, the majority was male (87%) and illness duration at time of scanning was on average 2.1 years (defined as time since first onset of psychotic symptoms). For further demographic and clinical information, see Table 1.

**Table 1:** **Demographic and clinical differences between three samples**.

	Sample A (*N* = 98)	Sample B (*N* = 146)	Sample C (*N* = 104)	Test statistics	*p*	Direction
Mean age, years (SD)	22.5 (3.1)	26.4 (5.8)	36.0 (11.1)	*F*(2,347) = 94.4	<0.001	A < B < C
Female/male (% male)	13/85 (87%)	28/118 (81%)	33/73 (69%)	χ^2^ (2) = 10.3	0.006	A and B < C
Mean GAF (SD)	35.1 (9.9)	51.7 (15.5)	52.3 (17.6)	*F*(2,305) = 44.4	<0.001	A < B and C
Mean illness duration (years) (SD)	2.1 (2.2)	3.8 (3.4)	14.7 (10.6)	*F*(2,342) = 124.3	<0.001	A and B < C
Mean number psychotic episodes (SD)	1.1 (0.3)	1.7 (1.1)	12.3 (30.1)	*F*(2,298) = 16.0	<0.001	A and B < C
Medication typical/atypical/both	10/81/3	9/101/3	10/35/40	χ^2^(4) = 93.2	<0.001	A and B < C
Drug abuse/dependence/both/none	25/25/4/44	18/32/13/83	–	χ^2^(3) = 9.49	0.023	A > B

#### Sample B

A total of 146 patients with a non-affective psychotic disorder (schizophrenia: *n* = 118, schizophreniform: *n* = 8, schizoaffective disorder: *n* = 19 and delusional disorder: *n* = 1) were selected from the baseline measurement of the *Genetic Risk and OUtcome of Psychosis* (GROUP) study. This naturalistic multi-center follow-up study with three assessments within a 6-year time-span started in 2004 (22). Patients recruited at the University Medical Centre Utrecht (UMCU) were included. Patients had a mean age of 26.4, the majority was male (81%), and illness duration at time of scanning was on average 3.8 years (defined as time since first onset of psychotic symptoms). For further demographic and clinical information, see Table 1.

#### Sample C

Sample C includes 104 patients with a schizophrenia spectrum disorder who took part in a 5-year longitudinal brain imaging study at the UMCU between 1995 and 2004 (23, 24). Patients were recruited from various in- and out-patient clinics and met the DSM-IV criteria for schizophrenia or schizophreniform disorder. Those with schizophreniform disorder met the criteria for the diagnosis of schizophrenia after 1 year of illness. Substance dependence or abuse 6 months prior to inclusion led to exclusion of the study. For the current study, baseline information was used, except when the clinical information was insufficient. In that case, when possible, information from follow-up was used. Patients were of a mean age of 36.4 years, the majority was male (70%) and illness duration at time of scanning was on average 14.7 years (defined as time since first onset of psychotic symptoms). For demographic and clinical variables of the current sample, see Table 1.

Patients were not included when information from the CASH or clinical records was insufficient to obtain information on the lifetime presence of FRS, scans were of poor quality, or processing of the MRI scan was unsuccessful, leading to the exclusion of 15, 21, and 19 scans from sample A, B, and C respectively. All numbers presented in tables and figures are those after exclusion of these subjects.

Each sample was divided in two groups based on the presence of FRS. The FRS+ group consisted of patients who had experienced at least one FRD or FRH life-time or present-state according to the CASH. When this was not the case, patients were part of the FRS−group.

### Imaging

Magnetic resonance images of the brain were acquired. Sample A was scanned on a Philips Intera 3T. A 3-dimenisonal T1-weighted image was acquired with an 8-channel SENSE head coil. Images were acquired in a clinical setting. Consequently, TEs and TRs range between 4.585–4.604 ms and 9.735–9.833 ms, respectively. Furthermore, flip angle = 8°, FOV = 256 × 256 and slice thickness = 1.2 mm. Sample B was scanned on a Philips Achieva 1.5T, SENSE 6 channel, TE = 4.6 ms, TR = 30 ms, flip angle = 30°, FOV = 256 × 256 and slice thickness = 1.2 mm. Sample C was acquired on a Philips Intera 1.5T, with TE = 4.6 ms, TR = 30 ms, flip angle = 30°, FOV = 256 × 256, and slice thickness = 1.2 mm.

Post processing of sample A was done on the e-Bioinfra Gateway (25), a web application that provides facilitated access to the Dutch Grid infrastructure to analyze large data collections. Samples B and C were processed on the neuroimaging computer network at the Department of Psychiatry, UMCU, Utrecht. All images were coded to ensure investigator blindness to subject identification and diagnosis.

Cortical and subcortical reconstruction and volumetric segmentation was performed with the Freesurfer image analysis suite v5.1.0, which is documented and freely available for download online^1^. Processing of a volumetric T1-weighted image includes automated Talairach transformation, intensity normalization (26), removal of non-brain tissue (27), segmentation of the subcortical white matter and deep gray matter volumetric structures, including accumbens, amygdala, caudate, hippocampus, pallidum, putamen, and thalamus (28, 29), tessellation of the gray matter white matter boundary, and automated topology correction (30–33). Next, the surface is inflated (32) for registration to a spherical atlas, which utilizes individual cortical folding patterns to match cortical geometry across subjects (32). This is used to parcellate the cerebral cortex into a map with units based on gyral and sulcal structure (34). A total of 68 cortical ROIs (left and right ROI were summed to represent total volume) and 7 subcortical ROIs (thalamus, hippocampus, amygdala, caudate, accumbens, pallidum, and putamen) were included in this study. The maps produced are not restricted to the voxel resolution of the original data, thus are capable of detecting submillimeter differences.

All segmentations were visually checked using the guidelines and scripts of the ENIGMA project^2^ (35). Pial surface segmentations were visually checked and, when needed, manually corrected for mislabeling of tissues prior to analysis. Specific attention was put on the inspection of delineation of the cortical gray and white matter tissues. Parts mislabeled as gray matter (e.g., remaining skull/dura mater) were removed. Further, mapping of the ROIs and subcortical structures was assessed by visually checking internal slice and external 3D labeling. Finally, an outlier analysis for each ROI was done to indicate further visual checking for possible mislabeling. An ROI is labeled as an outlier when the ROI value is higher or lower than the group average ROI by multiplying the standard deviation with 2.698, leading to a 0.99 confidence interval. This was done with project R version 3.0.1, a free software environment for statistical computing and graphics available for download online^3^.

### Statistical analysis

All analyses were performed using the Statistical Package for Social Sciences (SPSS) version 20.0 for Windows (SPSS Inc., Chicago, IL, USA).

#### Demographics

First, we compared the three samples on age, gender, GAF score, illness duration, number of psychotic episodes with one-way analyses of variance (ANOVA), and χ^2^ tests. Second, similar analyses were performed within each sample to compare patients with and without FRS.

#### Group Comparison on Brain Measures

FreeSurfer processing of the brain images generated volumes of cortical and subcortical ROIs for each individual subjects. Within each sample, patients with and without FRS were compared on each ROI using univariate GLM. ROI volume was included as dependent variable and group (FRS+/FRS−) entered the analysis as independent variable. Age and gender were added as covariates. When significant ROI differences between patients with and without FRS are found, it was investigated if the effect in local cortical volume was explained by cortical thickness or surface area of this ROI. Since there is an uneven distribution of the number of patients per FRS group within the samples, Levene’s test was used to check for homogeneity of variances. To correct for multiple comparisons False Discovery Rate (FDR) was controlled at 0.05 (36). In addition, we report findings with an uncorrected *p* < 0.01.

## Results

### Demographics

As expected, the three samples differed significantly on age, illness duration, and number of psychotic episodes (all significantly higher in sample C, except for age where all groups differed significantly from one another). In addition, significant differences were found for gender ratio (significantly more females in sample C), GAF score (significantly lower in sample A), and current antipsychotic medication use (significantly more patients on typical antipsychotics in sample C). In addition, intracranial volume (ICV) did not differ significantly between FRS+ and FRS−.

Table 2 shows the comparison of demographic and clinical variables between patients with and without FRS within each sample. No significant differences were found between FRS+ and FRS− on any of the demographic and clinical variables.

**Table 2:** **Demographic and clinical differences between patients with and without FRS in all three samples**.

	Sample A (*N* = 98)				Sample B (*N* = 146)				Sample C (*N* = 104)			
	FRS+ (*n* = 63)	FRS− (*n* = 35)	Test statistics	*p*	FRS+ (*n* = 129)	FRS− (*n* = 17)	Test statistics	*p*	FRS+ (*n* = 96)	FRS− (*n* = 8)	Test statistics	*p*
Mean age, years (SD)	22.3 (3.1)	23.0 (3.0)	*F*(1,97) = 2.6	0.31	26.5 (5.7)	25.6 (6.1)	*F*(1,144) = 0.40	0.53	35.9 (10.9)	38.1 (13.9)	*F*(1,105) = 0.28	0.60
Female/male (% male)	9/54 (86%)	4/31 (89%)	χ^2^(1) = 0.16	0.69	26/103 (80%)	2/15 (88%)	χ^2^(1) = 0.68	0.41	30/68 (44%)	3/5 (63%)	χ^2^(1) = 0.16	0.67
Mean GAF (SD)	34.3 (9.2)	36.5 (11.0)	*F*(1,97) = 1.1	0.30	51.6 (15.8)	52.4 (13.4)	*F*(1,148) = 0.04	0.85	52.3 (17.3)	52.0 (23.1)	*F*(1,84) = 0.00	0.97
PANSS remission criteria met/not met	6/57	8/27	χ^2^(1) = 3.3	0.07	–	–	–	–	–	–	–	–
Mean PANSS positive scale (SD)	–	–	–	–	15.5 (5.8)	13.6 (4.8)	*F*(1,88) = 1.62	0.20	14.4 (5.4)	14.8 (5.2)	*F*(1,99) = 0.03	0.86
Mean PANSS negative scale (SD)	–	–	–	–	15.4 (5.5)	16.5 (5.8)	*F*(1,88) = 0.51	0.48	14.6 (6.4)	15.0 (3.3)	*F*(1,99) = 0.03	0.86
Mean PANSS psychopathologic scale (SD)	–	–	–	–	31.7 (8.9)	32.3 (7.3)	*F*(1,84) = 0.07	0.79	30.0 (10.6)	32.6 (9.0)	*F*(1,99) = 0.87	0.35
Mean illness duration (SD)	2.4 (2.1)	1.7 (2.2)	F(1,97) = 2.1	0.15	4.0 (3.4)	2.9 (2.9)	*F*(1,141) = 1.50	0.22	14.7 (10.6)	15.4 (13.6)	*F*(1,104) = 0.04	0.85
Mean number psychotic episodes (SD)	1.1 (0.4)	1.1 (0.2)	F(1,97) = 0.3	0.60	1.7(1.1)	1.6 (0.8)	*F*(1,149) = 0.14	0.71	13.4 (31.4)	1.2 (0.4)	*F*(1,56) = 0.74	0.39
Medication *typical/Atypical/both*	6/55/1	4/26/2	χ^2^(2) = 1.7	0.42	8/90/3	1/10/0	χ^2^(2) = 0.35	0.84	10/33/36	0/2/4	χ^2^(2) = 1.57	0.46
Drug abuse/dependence/both/none	18/13/3/29	7/12/1/15	χ^2^(3) = 2.5	0.47	15/29/12/71	3/3/1/10	χ^2^(3) = 0.8	0.84	–	–	–	–

### Group comparison on brain measures

**Figure 1:**
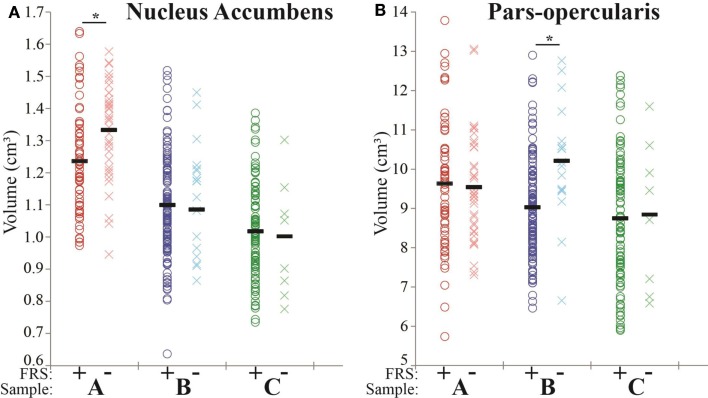
**On the *X* axis the FRS+ (dark-colored circles) and FRS− (light-colored crosses) groups are displayed for each sample (A, red; B, blue; and C, green)**. The Y axis represents the total volume (cm^3^). *Indicates FDR correct significant differences. **(A)** In sample A, volume of the nucleus accumbens is significantly smaller in FRS+ as compared with FRS−. **(B)** In sample B, volume of the pars-opercularis cortex is significantly smaller in FRS+ as compared with FRS−.

Levene’s homogeneity tests were not significant, suggesting equal variances in all ROIs between the FRS groups in all three samples. In sample A, a smaller nucleus accumbens was found in FRS+ relative to FRS−. In sample B, a smaller volume of pars-opercularis cortex was found in FRS+ patients as compared to FRS−. See Figure 1. No further significant differences were found in cortical and subcortical volumes between FRS+ and FRS− after FDR correction. Table 3 shows the results that reached the uncorrected *p* < 0.01. *Post hoc* analysis of the pars-opercularis in sample B showed that cortical surface area in FRS+ (3.08 cm^2^) was smaller than in FRS− (3.38 cm^2^; *F*(1,139) = 9.38 *p* = 0.003). Findings did not change when adding ICV or illness duration as covariate. Also, excluding patients with substance induced psychotic disorder, delusional disorder, and schizoaffective disorder did not change our findings.

**Table 3:** **Brain volume differences between patients with and without FRS in all three samples**.

	Sample A				Sample B				Sample C			
	FRS+ (*n* = 63)	FRS− (*n* = 35)	*F*	*p*	FRS+ (*n* = 129)	FRS− (*n* = 17)	F	*p*	FRS+ (*n* = 96)	FRS− (*n* = 8)	*F*	*p*
*Cortical*; pars-opercularis (mean volume ± SD cm^3^)	9.54 ± 0.18	9.59 ± 0.25	0.03	0.88	9.06 ± 0.11	10.18 ± 0.30	11.99	**0.0007**	8.74 ± 0.15	9.00 ± 0.540	0.21	0.64
(Mean surface area ± SD cm^2^)	3.08 ± 0.55	3.09 ± 0.74	0.01	0.91	3.08 ± 0.32	3.38 ± 0.92	9.39	**0.0026**	3.11 ± 0.47	3.18 ± 0.16	0.17	0.68
(Mean thickness ± SD cm)	0.55 ± 0.04	0.55 ± 0.05	0.006	0.94	0.53 ± 0.01	0.53 ± 0.08	0.05	0.82	0.51 ± 0.01	0.52 ± 0.02	0.10	0.76
*Subcortical*; nucleus accumbens (mean volume ± SD cm^3^)	1.24 ± 0.20	1.34 ± 0.27	8.82	**0.0037**	1.10 ± 0.13	1.10 ± 0.36	0.03	0.85	1.02 ± 0.15	1.00 ± 0.510	0.10	0.76

*Significant findings in bold; Cortical thickness *p* < 0.0015, Subcortical *p* < 0.0071, surface and thickness: *p* < 0.025*.

## Discussion

The aim of this study was to determine whether patients with first rank symptoms (FRS) differ in cortical and subcortical volumes from patients without these symptoms. The main finding is the lack of volume differences in the previously reported areas between these groups. This is consistent with previous research demonstrating in a study of 71 patients with schizophrenia that the severity of FRS showed no association with brain volume (18).

The only findings that reached statistical significance were a smaller nucleus accumbens and pars-opercularis cortex in patients with FRS as compared with those without FRS, in sample A and B respectively. *That significant group differences were not replicated between the three sample may indicate that these results are random, although the areas we found do have relevance for psychosis in general, and FRS in particular*. The nucleus accumbens, as part of the mesolimbic dopamine circuit, has been associated with reward, attention, and salience (37, 38), all abnormal in patients with psychotic symptoms. Abnormal dopaminergic signaling is associated with misattributing salience to stimuli and prediction errors, leading to positive symptoms, e.g., delusional beliefs such as FRD (38, 39). In addition, the pars-opercularis is part of Broca area, which plays a major role in semantic processes. Abnormalities in this area have been associated with the presence of auditory hallucinations (40), which are part of FRS. Here, we showed that the smaller pars-opercularis volume in patients with FRS could be explained by a smaller surface. Usually, abnormal surface is assumed to have a neurodevelopmental origin. As far as we know, there is no evidence that abnormalities in neurodevelopment lead to the emergence of specifically FRS. However, it remains unclear why volume decreases in the nucleus accumbens or pars-opercularis are only present in one of the included samples. The samples included in this study did differ significantly on several potentially relevant variables, such as illness duration, age, and outcome.

In previous studies, we compared the patient samples that are included in the current study to age- and gender-matched controls samples. The reported brain volume abnormalities are conform findings from a comprehensive meta-analysis integrating studies comparing brain volumes between (medication naive) schizophrenia patients to controls (41). That is, in sample A case-control differences showed smaller volumes of the amygdala, putamen, insula, para-hippocampus, and fusiform gyrus in patients with a non-affective psychotic disorder as compared with controls (42); in sample B case-control differences showed smaller volumes of the whole brain and cerebral white matter, increased volumes of the third and lateral ventricles, as well as local areas of cortical thinning, and reduced gray matter density in patients with schizophrenia as compared with controls (43), and finally in sample C patients demonstrated volume decreases in whole brain, cerebral gray matter, prefrontal gray and whiter matter volumes as well as volume increases in the lateral and third ventricles and peripheral CSF in schizophrenia patients as compared with controls (23, 44).

Previous studies suggested that smaller volumes of the (inferior) partial lobule, para-hippocampal gyrus, frontal cortex, cingulate gyrus, basal ganglia, and thalamus were associated with the presence of severity of FRS (14, 17). In these studies, it cannot be ruled out that the presence and severity of symptoms other than FRS are responsible for the association. Indeed, correlations between structural brain abnormalities and any one of the three main symptom dimensions, i.e., negative, positive, and disorganization, have been reported (45–48). For example, Maruff et al. (15) showed that patients with FRS had more severe symptoms of reality distortion as compared to patients without FRS. This might implicate that the volume reduction in parietal and frontal area is not completely due to the presence of FRS *per se*, but could also be explained by disorganization symptoms. Such symptoms have indeed an effect on total brain and cerebellar volume (46). Additionally, the severity of positive symptoms (which FRS are part of) have been found to correlate with reduced volumes in frontal and temporal regions (45), implicating that it is unclear what specific type of positive symptoms (e.g., FRS or paranoid delusions) may be responsible for the volume reduction. However, it is important to note that the reported correlation coefficients between cortical measures and symptom severity are generally low, suggesting that symptom severity explain only a small part of the variation in brain volume. In our study, we investigated whether patients with and without FRS differed on any of the other symptom measures or on number of psychotic episodes. This was not the case in either one of the three samples. This suggests that the few volume differences between FRS+ and FRS we found (e.g., smaller nucleus accumbens and pars-opercularis in FRS patients) cannot be explained by these clinical differences between the groups.

*Nevertheless, our finding of a subtle relationship between structural brain abnormalities and FRS does not exclude a stronger association between abnormal brain functioning and FRS*. Functional imaging studies have found a relationship between increased regional cerebral blood flow (rCBF) and severity of FRS (49, 50). In addition, schizophrenia patients with FRH, e.g., auditory hallucinations, consistently show functional abnormalities in speech and language brain areas and dysfunction of verbal memory systems during hallucinations (for an elaborate overview see (51). Also, cognitive functioning has been found associated with the presence and severity FRS. Patients, in particularly those with FRH, show impairments in source monitoring or source attribution (52, 53).

Our findings need to be interpreted taking several limitations into account. First, most of the patients in all three samples used antipsychotic medication at time of MRI scanning. Only information on type of medication (typical/atypical/both) was available. Therefore, we were not able to investigate the effect of life-time dose on the association between FRS and brain volumes. Second, in each of the three samples, patients with FRS were overrepresented (64% in sample A, 86% in sample B and 94% in sample C) leading to large differences in group size when performing group comparison. However, this distribution is largely in line with incidences reported in the literature (1–4).

Another limitation may be that FRS as a concept is broad and possibly consist of symptoms with different underlying brain pathology. Previously, we (and others) showed that FRS consist of two interrelated clusters of positive symptoms that interfere with boundaries of self and other (54–56), i.e., a cluster of auditory hallucinations (FRH, i.e., audible thoughts, conversational voices, and voices commenting on one’s actions) and a cluster of passivity delusions (*FRD*, i.e., thought withdrawal, thought broadcasting, thought insertion, and beliefs that impulses and/or actions are controlled by an outside force). Imaging studies that focused specifically on FRH demonstrated reduced gray matter volumes in sensory regions (51), a thinner cortex in the pars orbitalis, para-central lobule, fusiform gyrus, and inferior temporal gyrus (57) in patients with auditory hallucination as compared to controls and non-schizophrenia hallucinators. Also abnormal brain measures have been found in patients with FRD as compared to those without, albeit in different areas, i.e., reduced cortical volume in parietal and frontal areas (15), increased fractional anisotropy in the frontal cortex, cingulate gyrus, and basal ganglia, and decreased fractional anisotropy in the thalamus (17).

In conclusion, first rank symptoms have since long lost their diagnostic relevance for psychotic disorders in general and schizophrenia in particular. Their presence is associated with a number of specific cognitive and functional brain abnormalities, e.g., differences in language areas or deficits in sense of agency, suggesting that specific neural abnormalities may underlie FRS. However, we found only subtle volume decreases in the accumbens and pars-opercularis, which were inconsistent between three independent samples.

## Conflict of Interest Statement

The authors declare that the research was conducted in the absence of any commercial or financial relationships that could be construed as a potential conflict of interest.

## Footnotes

^1^http://surfer.nmr.mgh.harvard.edu

^2^http://enigma.ini.usc.edu/

^3^http://www.r-project.org/
